# How Prosocial Motivation Is Related to Nurses’ Taking Charge at Work: A Multiwave, Multisource Survey and Two Cross‐Sectional, Cross‐National Surveys

**DOI:** 10.1155/jonm/8090132

**Published:** 2026-07-13

**Authors:** Liping Zhou, Xiangyu Gao, Jessie Lin Wang, Shuji Pan, Tingting Chen, Yue Zhu, Jie Wang

**Affiliations:** ^1^ Nursing Department, Sir Run Run Shaw Hospital, School of Medicine, Zhejiang University, Hangzhou, China, zju.edu.cn; ^2^ Department of Management, Faculty of Business, Lingnan University, Hong Kong, China, ln.edu.hk; ^3^ School of Business Administration, Zhejiang Gongshang University, Hangzhou, China, zjgsu.edu.cn; ^4^ Nottingham University Business School China, University of Nottingham Ningbo China, Ningbo, China, nottingham.edu.cn

**Keywords:** general job reflective learning, nurse, prosocial motivation, taking charge, work overload

## Abstract

**Background:**

Prosocial motivation drives nurses to help patients, yet contemporary healthcare also requires proactive, change‐oriented behaviors such as taking charge. How prosocial motivation translates into taking charge remains poorly understood.

**Aim:**

To investigate how and why prosocial motivation is related to nurses’ taking charge and how work overload moderates this process.

**Design:**

Study 1 employed a multiwave, multisource survey design. Studies 2 and 3 further tested the hypotheses using cross‐sectional, cross‐national designs.

**Methods:**

In Study 1, data were collected in a general hospital in China at three time points (two weeks apart) from 275 nurses and their immediate supervisors. Studies 2 and 3 further utilized cross‐sectional designs, recruiting 636 and 441 nurses, respectively, from multiple countries via Prolific. Descriptive analyses, confirmatory factor analyses, and mediation and moderated mediation analyses were performed.

**Results:**

Nurses’ prosocial motivation was positively related to taking charge, mediated by general job reflective learning. Work overload moderated the positive association between prosocial motivation and reflective learning, and the indirect effect of prosocial motivation on taking charge via reflective learning was weaker under high work overload.

**Conclusion:**

Prosocial motivation was positively associated with nurses’ general job reflective learning, which in turn related to their taking charge. This highlights that nurse proactivity is not merely a spontaneous act of goodwill but a deliberate, self‐regulated process. Moreover, the positive association between prosocial motivation and reflective learning was weaker under high workload. This suggests that prosocial motivation alone may not translate into taking charge when nurses lack the necessary cognitive resources needed for self‐regulated learning.

**Implications for Nursing Management:**

This research provides a clear mandate for healthcare administrators: to harness the vast potential of their nursing workforce to advance patient care and organizational quality, organizations must not only cultivate prosocial motivation but also actively support reflective learning practice. Most critically, they must manage workloads to ensure nurses have the resources required to translate their prosocial motivation into effective action.

## 1. Introduction

Nurses, the largest segment of the healthcare workforce, serve on the frontline of care delivery [1]. In this mission‐driven profession [2], prosocial motivation, defined as the desire to expend effort to benefit other people through one’s work [3], has been identified as an important disposition. This motivation drives nurses to help patients [4, 5]. However, in today’s increasingly uncertain and complex healthcare landscape—marked by rapid technological advancements, recurring global public health crises, and diverse patient needs [6, 7]—routine helping may not be sufficient to adapt to these changes and ensure a sustainable healthcare system. In such contexts, proactive behaviors, such as taking charge, which is defined as employees’ voluntary and constructive efforts to implement improvements in work methods, policies, or procedures [8], are widely acknowledged by professional healthcare committees as essential to modern nursing [9–11]. Nurses’ direct patient‐care experiences enable them to identify problems and implement improvements that benefit patients and the broader healthcare environment [11].

Despite its potential value, taking charge in nursing remains a risky endeavor. Evidence suggests that people who attempt to initiate change often face resistance from colleagues or even reprimand from supervisors [12, 13]. Moreover, healthcare institutions tend to reserve change‐oriented initiatives for formal leadership roles such as charge nurses [14, 15], raising questions about how frontline nurses navigate the constraints of hierarchical systems. Consequently, although prosocial motivation may inspire nurses to improve outcomes for others [16], the translation of this motivation into proactive, change‐oriented behavior is neither straightforward nor well understood. This ambiguity underscores the need to examine whether, why, and under what conditions nurses materialize prosocial motivation into taking charge.

Because taking charge is a deliberate decision process [8], self‐regulation theory, which comprises self‐generated thoughts, feelings, and actions that are planned and adapted to attain personally valued goals [17], provides an ideal framework to examine the self‐regulated cognitive process linking prosocial motivation and taking charge. Building on research on prosocial motivation and self‐regulation [3, 16, 17], we propose that general job reflective learning—the process of consciously evaluating past work experiences to guide future behaviors [18]—serves as a key self‐regulatory mechanism that translates nurses’ prosocial motivation into taking charge. Because prosocially motivated employees view work as a means to the end goal of benefiting others, this orientation elicits self‐regulatory efforts to improve others’ outcomes [3]. A key self‐regulatory process involves reflecting on the present situation relative to desired outcomes [19]. Nurses with high prosocial motivation have a strong desire to improve patients’ welfare (desired outcomes), which drives them to actively learn from experience to prevent harm and improve care efficiency. Reflective learning is a change‐oriented mechanism that centers on reappraising experiences to identify alternatives and areas for improvement [20]. Through reflecting on and learning from experience, nurses systematically analyze complex clinical and organizational situations, identify strengths and weaknesses of current practices, and generate potential solutions based on learned insights. This process enhances their job‐specific knowledge, strategic thinking, and confidence [18, 21], which facilitates taking charge [22]. In short, general job reflective learning transforms prosocial intent into a well‐informed and credible plan for constructive change, thereby enabling nurses to demonstrate proactivity in an informed and risk‐aware way [23].

**FIGURE 1:**
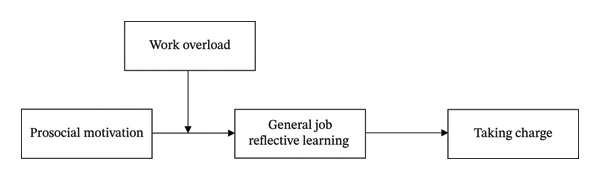
The hypothesized model.

Because effective self‐regulation requires sufficient resources [24], we further theorize a critical boundary condition that constrains resource availability in nursing: work overload. In the demanding clinical environment, work overload represents a critical contextual stressor [25, 26]. The global nursing shortage and high patient‐to‐nurse ratios documented by the WHO [1] confirm that excessive workload is a pervasive reality. Work overload can translate nurses’ internal desire to benefit patients into external pressure, leading to heightened stress and fewer resources for self‐regulatory efforts [3, 27]. Because reflective learning is a complex, effortful cognitive process that requires substantial resources [18], prosocially motivated nurses under high work overload tend to prioritize immediate task demands and reactive problem‐solving over discretionary, future‐oriented activities like reflective learning. Consequently, the positive indirect effect between prosocial motivation and taking charge via general job reflective learning is weaker under high work overload. Figure 1 presents our research model.

This research makes several important contributions. First, it extends the prosocial motivation literature by demonstrating that nurses’ desire to benefit others can motivate proactive efforts to challenge the status quo and drive systemic improvements in the healthcare environment. By shifting attention from formal leadership roles to frontline nurses as initiators of change, this study highlights microfoundations of organizational improvement. Second, it enriches the integration of prosocial motivation and proactivity literature by specifying general job reflective learning as a crucial, yet understudied, cognitive mechanism that bridges prosocial motivation and proactive behavior. By revealing this critical self‐regulated learning process, this research answers the call of investigating how employees act proactively in an informed and risk‐aware manner [23]. Third, it contributes to the workload literature by examining the role of work overload from a cognitive perspective. Unlike previous research that primarily focuses on the negative consequences of workload for health and well‐being, we propose that under excessive workload, prosocially motivated employees may have diminished capacity to engage in reflective learning, which in turn is associated with a lower likelihood of taking charge. Finally, this research offers critical practical implications for healthcare administrators, highlighting the importance of fostering reflective learning practices and strategically managing workloads to enable nurses to translate their prosocial motivation into taking charge, thereby enhancing patient outcomes and overall organizational effectiveness in dynamic and complex healthcare settings.

## 2. Theory and Hypothesis Development

### 2.1. Prosocial Motivation and General Job Reflective Learning

Prosocial motivation is the desire to benefit others through one’s work [3]. In nursing, this motivation is central, as the core mission of the profession is patient advocacy and welfare [2, 5]. According to self‐regulation theory, individuals engage in forethought, performance control, and self‐reflection to attain valued goals, and this process is driven by personal (e.g., motives and self‐control) and environmental factors (e.g., work settings) [17]. Because prosocial motivation is other‐oriented and future‐focused, it elevates others’ welfare as a salient work goal and stimulates self‐regulatory efforts to achieve that goal [3, 28]. Reflective learning is a key process of self‐regulation: it involves intentionally reappraising work experiences to guide future behavior and is activated when individuals hold strong outcome standards and a desire to improve those outcomes [18]. Therefore, prosocial motivation strengthens the internal drive for reflective learning, shifting nurses from merely following protocols toward actively reflecting on how their practice connects to patients or team needs. Moreover, because prosocial motivation directs employees’ attention towards other‐related situational cues [28], it broadens the scope and deepens the depth of reflection, increasing the likelihood that they will identify discrepancies between current and desired outcomes. To reduce these discrepancies, prosocially motivated employees are more likely to deploy reflective learning to analyze past actions, extract lessons, and plan improved future responses [18, 20]. In conclusion, nurses with high prosocial motivation have a strong desire to improve patients’ welfare and are sensitive to patients’ needs. Consequently, they are more likely to recognize discrepancies between intended outcomes (e.g., patient comfort) and actual outcomes (e.g., persistent distress), which promotes them to reflect on previous practice for improvements (reflective learning). Thus, by elevating others’ welfare as a guiding standard and energizing discrepancy‐reduction efforts, prosocial motivation should spur more frequent and deeper general job reflective learning.

**Hypothesis 1.**

Prosocial motivation is positively related to general job reflective learning.

### 2.2. The Mediating Role of General Job Reflective Learning

We propose that general job reflective learning serves as the critical self‐regulatory mechanism translating prosocial motivation into taking charge. Taking charge entails discretionary, constructive efforts to change work methods, policies, or procedures and requires calculated and deliberate decision‐making about whether change is feasible and valuable [8]. While prosocial motivation increases employees’ felt responsibility for bringing positive change to others [3], employees also require informed plans, confidence, and credible solutions to produce effective change [23].

Reflective learning is a key self‐regulation process through which employees interpret work experience and update strategies to improve future performance [17]. Through reflecting on and learning from experience, nurses systematically assess current practices’ strengths and limitations, identify root causes of problems, and develop potential solutions based on learned insights. This process enhances their job‐specific knowledge, strategic thinking, and confidence [18, 21]. For example, when a nurse reflected on a failure to initiate an emergency procedure, the reflection revealed that the procedure was overly complex. This insight enabled the nurse to propose an adapted, simplified procedure—an instance of taking charge [18]. Accumulating research shows that reflective learning enables change‐oriented behaviors [21, 29]. In sum, general job reflective learning transforms prosocial motivation into a well‐informed and strategically sound plan of action, significantly increasing the likelihood of initiating and sustaining constructive change.

**Hypothesis 2.**

General job reflective learning mediates the relationship between prosocial motivation and taking charge.

### 2.3. The Moderating Role of Work Overload

While motivation can initiate self‐regulatory efforts, the effectiveness of self‐regulation also depends on situational factors that are related to individuals’ capacity and resource availability [17, 24]. In nursing, work overload—characterized by excessive patient assignments, administrative burden, and extended shifts—is a pervasive stressor that significantly depletes time and cognitive resources and impairs individuals’ capacity to manage their work processes [25, 27]. Crucially, such pressures are not uniform. Even within the same hospital, nurses face varying levels of workload due to differences in clinical units, shift patterns, patient acuity, and personal experience [30, 31], which involves meaningful individual‐level variance in work overload. We propose that under high work overload, the positive relationship between prosocial motivation and general job reflective learning is weaker, as prosocially motivated nurses may have diminished capacity and intention to engage in reflection.

High work overload can make nurses feel overwhelmed and experience a diminished sense of control [27]. Under these conditions, nurses high in prosocial motivation face heightened stress associated with satisfying patients’ needs [3], which makes them expend more self‐regulatory resources to manage their responses to overload. This greater resource expenditure leads to additional resource losses that further impair their capacity to focus attention and sustain effort on complex cognitive activities [24]. Given that general job reflective learning is an effortful and resource‐intensive cognitive activity that demands focused attention, time for introspection, and psychological space to process complex information [18, 32], nurses high in prosocial motivation may find it particularly challenging to initiate and sustain such learning under high work overload. Moreover, high work overload can lead to an attention shift toward reactive, immediate task‐focused behaviors [24]. Because prosocial nurses are highly sensitive to situational cues [3], they are more likely to notice and respond to patients’ immediate needs during overload, further limiting the time and cognitive resources available for reflective learning. For instance, when handling multiple trauma cases (work overload), nurses motivated to ensure patients’ health (prosocial motivation) may focus entirely on current tasks (e.g., administering medications, stabilizing patients) rather than reflecting on past work experience.

Conversely, when work overload is lower, prosocially motivated nurses experience greater control over how they help patients and have more time and cognitive resources available to pursue their prosocial goals. To foster future improvements [3], prosocial nurses in lower work overload contexts are more likely to engage in self‐regulatory efforts such as reflection, after‐action reviews, and cross‐case learning, thereby strengthening the positive relationship between prosocial motivation and general job reflective learning.

**Hypothesis 3.**

Work overload moderates the relationship between prosocial motivation and general job reflective learning, such that this positive relationship is weaker when work overload is higher (rather than lower).

Building on H2 and H3, we further propose that work overload attenuates the entire indirect pathway from prosocial motivation to taking charge via general job reflective learning. High work overload, by depleting self‐regulatory resources and shifting attention to immediate demands, impedes the pathway from prosocial intent to reflective learning. As a result, the accrual of job knowledge, strategic insights, and efficacy that typically promotes taking charge is reduced. Under lower work overload, prosocial motivation can more readily translate into reflective learning and, consequently, into taking charge. For instance, a pediatric nurse with strong prosocial motivation typically reflects on how to improve vaccine administration comfort and proposes adjustments. However, during a staffing shortage (high work overload), the nurse spends 12 h completing direct care tasks (e.g., feeding, medication) and has no time and energy to reflect on vaccine administration. Without this reflective process, the nurse may be less likely to identify questions or propose changes, and the indirect association between prosocial motivation and taking charge is correspondingly weaker.

**Hypothesis 4.**

Work overload moderates the indirect relationship between prosocial motivation and taking charge via general job reflective learning, such that this indirect relationship is weaker when work overload is high (versus low).

### 2.4. Overview of Studies

To ensure the robustness and generalizability of our findings, this research employed a three‐study design. Study 1 was conducted in a hospital in China; Study 2 was conducted using a multinational sample to test whether the findings could be generalized to the broader nursing workforce; Study 3 aims to constructively replicate the findings and add robustness tests by controlling for proactive personality, a critical antecedent of taking charge [33]. By demonstrating consistent findings across three independent samples, we provide greater confidence in our conclusions.

#### 2.4.1. Analytical Approach

We performed all statistical analyses using Mplus 8. We conducted a series of confirmatory factor analyses (CFAs) to evaluate the distinctiveness of the four variables in the hypothesized model. To test the research model, we included all hypothesized relationships in integrative path models. To estimate the moderating effect, we grand‐mean‐centered prosocial motivation and work overload to reduce potential multicollinearity [34]. We examined indirect and conditional indirect effects using bias‐corrected bootstrap confidence intervals (BC CIs) based on 10,000 resamples [35].

#### 2.4.2. Ethical Considerations

Ethical approval for all three studies was obtained from the Academic Development and Ethics Committee, Zhejiang Gongshang University. Prior to participation, individuals were informed that their involvement in the study was voluntary, they may withdraw at any time, and their responses would be treated as strictly confidential.

## 3. Study 1 Method and Results

### 3.1. Design and Participants

Study 1 was conducted in a general hospital in China at three time points, with 2‐week intervals. A total of 450 nurses and their immediate supervisors were invited to take part in the study. At Time 1, nurses completed a questionnaire assessing their prosocial motivation, work overload, and demographic characteristics. At Time 2, the nurses reported their level of general job reflective learning. At Time 3, the nurses’ immediate supervisors evaluated the extent to which each nurse demonstrated taking charge. We also conducted interviews with the nurse managers to gain a better understanding of the nursing work context, including how nurses had varying workloads and how they took charge to improve work methods, policies, or procedures.

A total of 275 nurses and their immediate supervisors completed surveys over the three‐wave data collection process, resulting in a response rate of 61.11%. The participants had an average age of 35.45 years (SD = 7.16) and an average tenure in nursing jobs of 11.79 years (SD = 8.36). The sample consisted predominantly (95.3%) of female nurses. Demographic characteristics of the sample are presented in Table 1.

**TABLE 1:** Demographic characteristics of samples.

**Demographic characteristics**	**Study 1 (*N* = 275)**		**Study 2 (*N* = 636)**		**Study 3 (*N* = 441)**	
	**Frequency**	**Percentage (%)**	**Frequency**	**Percentage (%)**	**Frequency**	**Percentage (%)**
Gender						
Male	13	4.7%	124	19.5%	86	19.5%
Female	262	95.3%	512	80.5%	355	80.5%
Education level						
Doctoral degree	0	0.0%	5	0.8%	1	0.2%
Master’s degree	9	3.3%	137	21.5%	83	18.8%
Bachelor’s degree	261	94.9%	400	62.9%	287	65.1%
College diploma	4	1.5%	85	13.4%	59	13.4%
High school diploma or below	1	0.4%	9	1.4%	11	2.5%
Country of residence						
China	275	100%				
United Kingdom			259	40.7%	157	35.6%
United States			178	28.0%	151	34.2%
South Africa			88	13.8%	26	5.9%
Canada			26	4.1%	28	6.3%

	**Mean**	**SD**	**Mean**	**SD**	**Mean**	**SD**

Age	35.45	7.16	39.47	12.41	39.33	11.48
Job tenure	11.79	8.36	13.75	11.91	13.37	10.82

### 3.2. Measures

The survey was conducted in Chinese, and all items were translated using a standard back‐translation procedure to ensure accuracy.

#### 3.2.1. Prosocial Motivation (Time 1)

Prosocial motivation was measured by a four‐item scale (*α* = 0.89) developed by Grant [3]. Participants were asked a question: “Why are you motivated to do your work?” Items for prosocial motivation are “Because I care about benefiting others through my work,” “Because I want to help others through my work,” “Because I want to have a positive impact on others,” and “Because it is important to me to do good for others through my work.” The nurses responded to the items on a 7‐point Likert‐type scale (from 1 = *strongly disagree* to 7 = *strongly agree*).

#### 3.2.2. Work Overload (Time 1)

Work overload was measured by a three‐item scale (*α* = 0.80) from Yoon et al. [27]. Items are “I do not have enough time to get everything done on the job,” “I have to work very fast on the job,” and “The workload on my job is not heavy.” The nurses responded to the items on a 5‐point Likert‐type scale (from 1 = *strongly disagree* to 5 = *strongly agree*). Interviews with the nurse managers revealed considerable variance in workloads among the nursing staff. Even within the same hospital, interdepartmental variations are prominent; emergency and surgical units generally experience higher operational demands than other departments. Additionally, intradepartmental workloads vary due to the unpredictable nature of patient assignments, as the acuity levels and complex care needs of individual patients differ on a case‐by‐case basis.

#### 3.2.3. General Job Reflective Learning (Time 2)

General job reflective learning was measured by an eight‐item scale (*α* = 0.90) developed by Peltier et al. [20]. Sample items are “I often reappraised my experiences so I could learn from them” and “I often reflected on my actions to see whether I could improve them.” The nurses responded to the items on a 5‐point Likert‐type scale (from 1 = *strongly disagree* to 5 = *strongly agree*).

#### 3.2.4. Taking Charge (Time 3)

Supervisors evaluated their subordinates taking charge on a six‐item scale (*α* = 0.88) developed by Morrison and Phelps [8]. Although the original scale comprises 10 items, consultation with the nurse managers indicated that four items were not appropriate for capturing taking charge in this hospital’s work environment. In order to enhance the scale’s contextual relevance and measurement validity, we excluded these four items from the original measure. Specifically, the items adopted in this study are “This nurse often tries to change how his or her job is executed in order to be more effective,” “This nurse often tries to bring about improved procedures for the work unit,” “This nurse often tries to institute new work methods that are more effective for the work unit,” “This nurse often tries to correct a faulty procedure or practice,” “This nurse often tries to implement solutions to pressing problems in the work unit,” and “This nurse often tries to introduce new structures, technologies, or approaches to improve efficiency.” The supervisors responded to the items on a 7‐point Likert‐type scale (from 1 = *strongly disagree* to 7 = *strongly agree*). Although nurses must adhere to standard clinical protocols, nurse managers emphasized that there are ample opportunities for taking charge in daily practice, which is critical for nurses’ performance. For instance, triage nurses proactively label high‐risk patients with an orange tag after initial screening. This allows clinic nurses to quickly identify and reassess them, thus improving patient safety. Another example is that triage patients suspected of acute coronary syndrome (ACS) often present with dizziness, abdominal pain, or syncope instead of typical chest pain. Because stable patients without obvious conditions (e.g., gastrointestinal bleeding, epilepsy) bypass the resuscitation room, triage nurses proactively initiated an upfront ECG screening. This workflow redesign allows for the immediate detection of myocardial ischemia, preventing missed diagnoses in atypical cases and improving patient outcomes.

#### 3.2.5. Control Variables (Time 1)

Nurses’ age, gender, job tenure, and educational level were controlled in this study because they have been shown to be associated with individuals’ general job reflective learning and taking charge.

### 3.3. Confirmatory Factor Analyses

We performed a series of CFAs to test the distinctiveness of the four key variables in the study (i.e., prosocial motivation, work overload, general job reflective learning, and taking charge). The baseline four‐factor model had a good fit (*χ* ^2^ (183) = 357.17, CFI = 0.95, TLI = 0.95, SRMR = 0.05, RMSEA = 0.06). This model has a better fit compared to (1) a three‐factor model that combines work overload and prosocial motivation (Δ*χ* ^2^ (3) = 289.22, *p* < 0.01, CFI = 0.87, TLI = 0.86, SRMR = 0.08, RMSEA = 0.10); (2) a two‐factor model that combines work overload, prosocial motivation, and general job reflective learning (Δ*χ* ^2^ (5) = 979.52, *p* < 0.01, CFI = 0.68, TLI = 0.65, SRMR = 0.12, RMSEA = 0.15); and (3) a one‐factor model that combines all variables (Δ*χ* ^2^ (6) = 2071.43, *p* < 0.01, CFI = 0.38, TLI = 0.31, SRMR = 0.19, RMSEA = 0.21). These results support the distinctiveness of the four constructs, validating our measurement model for subsequent analyses.

### 3.4. Test of the Mediating Effects

Table 2 presents the descriptive statistics and Pearson correlation coefficients for the study variables. Because some supervisors rated more than one nurse, the data exhibited a partially nested structure. To account for potential nonindependence in parameter estimation, we applied the TYPE = COMPLEX option in Mplus. As Table 3 shows, prosocial motivation was positively related to general job reflective learning (*B* = 0.22, *p* < 0.01), providing support for Hypothesis 1. Furthermore, general job reflective learning was positively related to taking charge (*B* = 0.32, *p* < 0.01). Bootstrapping analyses revealed that the indirect effect of prosocial motivation on taking charge via general job reflective learning was positive (indirect effect = 0.07, 95% BC CI [0.02, 0.16]). These results support Hypothesis 2.

**TABLE 2:** Descriptive statistics, reliabilities, and Pearson correlation coefficients (Study 1).

Variables	1	2	3	4	5	6	7	8
1. Age								
2. Gender	0.10							
3. Education	−0.12^∗^	0.08						
4. Job tenure	0.98^∗∗^	0.12^∗^	−0.18^∗∗^					
5. Prosocial motivation	0.18^∗∗^	0.01	−0.01	0.18^∗∗^	**(0.89)**			
6. Work overload	−0.22^∗∗^	−0.07	−0.04	−0.22^∗∗^	−0.02	**(0.80)**		
7. General job reflective learning	0.08	0.01	0.04	0.08	0.37^∗∗^	−0.02	**(0.90)**	
8. Taking charge	0.05	0.01	0.10	−0.00	0.16^∗∗^	0.07	0.19^∗∗^	**(0.88)**
Mean					6.00	3.13	3.82	5.40
SD					0.87	0.86	0.52	0.91

*Note*: *N* = 275. Cronbach’s alphas are shown in bold on the diagonal.

^∗^ *p* < 0.05.

^∗∗^ *p* < 0.01.

**TABLE 3:** Mediation model and moderated mediation model (Study 1).

	Mediation model		Moderated mediation model	
	General job reflective learning	Taking charge	General job reflective learning	Taking charge
Control variable				
Age	−0.00	0.14^∗∗^	−0.00	0.14^∗∗^
Gender	−0.02	0.10	−0.02	0.10
Education	0.11	0.11	0.13	0.11
Job tenure	0.00	−0.12^∗∗^	0.00	−0.12^∗∗^
Independence variable				
Prosocial motivation	0.22^∗∗^		0.22^∗∗^	
Mediator				
General job reflective learning		0.32^∗∗^		0.32^∗∗^
Moderator				
Work overload			0.03	
Prosocial motivation × Work overload			−0.13^∗∗^	
*R* ^2^	0.14^∗^	0.10^∗^	0.17^∗^	0.10^∗^

*Note*: *N* = 275. Bootstrap sample size = 10,000.

^∗^ *p* < 0.05.

^∗∗^ *p* < 0.01.

### 3.5. Test of the Moderating Effect and the Moderated Mediation Effects

**FIGURE 2:**
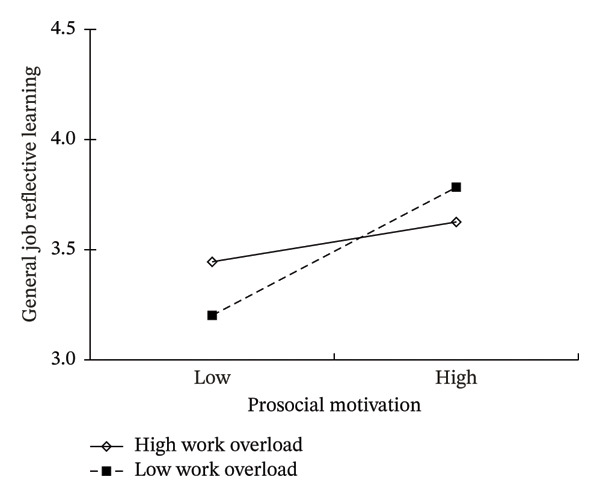
The interaction of prosocial motivation and work overload (Study 1).

Hypothesis 3 proposes that work overload moderates the relationship between prosocial motivation and general job reflective learning. As shown in Table 3, the interaction between prosocial motivation and work overload on general job reflective learning (*B* *=* −0.13, *p* *<* 0.01) was statistically significant. In addition, simple slope analyses revealed that at lower levels of work overload (−1 SD), the positive relationship between prosocial motivation and general job reflective learning was significant (simple slope = 0.33, *p < *0.01), while this relationship became nonsignificant at higher levels of work overload (+1 SD) (simple slope = 0.10, *p = *0.06). Thus, Hypothesis 3 was supported. The moderation effect is plotted in Figure 2.

Furthermore, we examined the moderated mediation effects. The index of the moderated mediation was negatively significant (index = −0.04, 95% BC CI = [−0.10, −0.01]) for the indirect relationship between prosocial motivation and taking charge mediated by general job reflective learning. The indirect relationship between prosocial motivation and taking charge was stronger at lower levels of work overload (indirect effect = 0.11, 95% BC CI = [0.03, 0.24]) than at higher levels of work overload (indirect effect = 0.03, 95% BC CI = [0.002, 0.11]). These results supported Hypothesis 4.

## 4. Study 2 Method and Results

### 4.1. Design and Participants

Study 2 tested the hypotheses using a cross‐sectional design with a cross‐national sample recruited from Prolific. Prolific is an online platform designed for recruiting participants for academic research. We applied Prolific’s prescreening function to filter participants who had indicated “Nurse” under the “Employment Sector within Medicine/Healthcare” category. Prolific identified a total of 1917 eligible nurses who had been active in the past 90 days. We distributed our survey to this pool. Out of the 658 participants who initiated the survey, 636 completed it, resulting in a 3% dropout rate. Each participant was compensated GBP 1.00 for a total of 5 min of compensable time.

Among the 636 participants, the average age was 39.47 years (SD = 12.41). The sample consisted predominantly of female nurses (80.5%). On average, participants reported a tenure in nursing jobs of 13.75 years (SD = 11.91). Their countries of residence were primarily the United Kingdom (40.7%) and the United States (28.0%), with the remainder distributed across other countries (e.g., South Africa and Canada). Demographic characteristics of the sample are presented in Table 1.

### 4.2. Measures

#### 4.2.1. Prosocial Motivation, Work Overload, General Job Reflective Learning, and Taking Charge

We used the same scales as in Study 1 to measure prosocial motivation, work overload, and general job reflective learning. Cronbach’s alphas were 0.92, 0.80, and 0.85, respectively.

For assessing taking charge, we used the original 10‐item scale (*α* = 0.91) developed by Morrison and Phelps [8]. We conducted a robustness check by adopting the six‐item scale used in Study 1, and the statistical conclusion remains unchanged.

#### 4.2.2. Control Variables

Consistent with Study 1, we included nurses’ age, gender, job tenure, and educational level as control variables in this analysis.

### 4.3. Confirmatory Factor Analyses

We conducted CFAs to assess the distinctiveness of the four variables in the hypothesized model. The baseline four‐factor model, including prosocial motivation, work overload, general job reflective learning, and taking charge, demonstrated a satisfactory fit to the data (*χ* ^2^ (269) = 1022.26, CFI = 0.91, TLI = 0.90, SRMR = 0.06, RMSEA = 0.07). This model provided a significantly better fit than the alternative models: (1) a three‐factor model that combines prosocial motivation and work overload (Δ*χ* ^2^ (3) = 661.71, *p* < 0.01, CFI = 0.83, TLI = 0.82, SRMR = 0.08, RMSEA = 0.09); (2) a two‐factor model that combines prosocial motivation, work overload, and general job reflective learning (Δ*χ* ^2^ (5) = 1697.19, *p* < 0.01, CFI = 0.71., TLI = 0.69, SRMR = 0.11, RMSEA = 0.12); and (3) a single‐factor model (Δ*χ* ^2^ (6) = 2994.16, *p* < 0.01, CFI = 0.56, TLI = 0.51, SRMR = 0.12, RMSEA = 0.15). These results lend strong support for the distinctiveness of the four variables in the hypothesized model.

### 4.4. Test of the Mediating Effects

Table 4 displays descriptive statistics, reliabilities, and Pearson correlation coefficients among the examined variables. The results in Table 5 indicate that prosocial motivation was positively related to general job reflective learning (*B* = 0.35, *p* < 0.01), thereby supporting Hypothesis 1.

**TABLE 4:** Descriptive statistics, reliabilities, and Pearson correlation coefficients (Study 2).

Variables	1	2	3	4	5	6	7	8
1. Age								
2. Gender	0.07							
3. Education	−0.04	−0.04						
4. Job tenure	0.84^∗∗^	0.13^∗∗^	−0.01					
5. Prosocial motivation	0.03	0.07	−0.04	0.06	**(0.92)**			
6. Work overload	0.02	0.03	−0.01	0.02	−0.07	**(0.80)**		
7. General job reflective learning	−0.11^∗∗^	0.01	0.06	−0.09^∗^	0.50^∗∗^	0.01	**(0.85)**	
8. Taking charge	0.05	0.01	0.18^∗∗^	0.08^∗^	0.39^∗∗^	0.00	0.52^∗∗^	**(0.91)**
Mean					6.26	3.74	4.00	5.13
SD					0.77	0.87	0.53	1.00

*Note: N* = 636. Cronbach’s alphas are shown in bold on the diagonal.

^∗^ *p* < 0.05.

^∗∗^ *p* < 0.01.

**TABLE 5:** Mediation model and moderated mediation model (Study 2).

	Mediation model		Moderated mediation model	
	General job reflective learning	Taking charge	General job reflective learning	Taking charge
Control variable				
Age	−0.00	0.00	−0.00	0.00
Gender	−0.01	−0.04	−0.01	−0.04
Education	0.07^∗^	0.23^∗∗^	0.07^∗∗^	0.23^∗∗^
Job tenure	−0.00	0.01	−0.00	0.01
Independence variable				
Prosocial motivation	0.35^∗∗^	0.22^∗∗^	0.36^∗∗^	0.22^∗∗^
Mediator				
General job reflective learning		0.82^∗∗^		0.82^∗∗^
Moderator				
Work overload			0.03	
Prosocial motivation × Work overload			−0.08^∗^	
*R* ^2^	0.27^∗∗^	0.33^∗∗^	0.29^∗∗^	0.33^∗∗^

*Note: N* = 636. Bootstrap sample size = 10,000.

^∗^ *p* < 0.05.

^∗∗^ *p* < 0.01.

Furthermore, general job reflective learning was positively related to taking charge (*B* = 0.82, *p* < 0.01). Bootstrapping results show that the indirect effect of prosocial motivation on taking charge through general job reflective learning was significant (indirect effect = 0.29, 95% BC CI = [0.21, 0.37]), supporting Hypothesis 2.

### 4.5. Test of the Moderating Effect and the Moderated Mediation Effects

**FIGURE 3:**
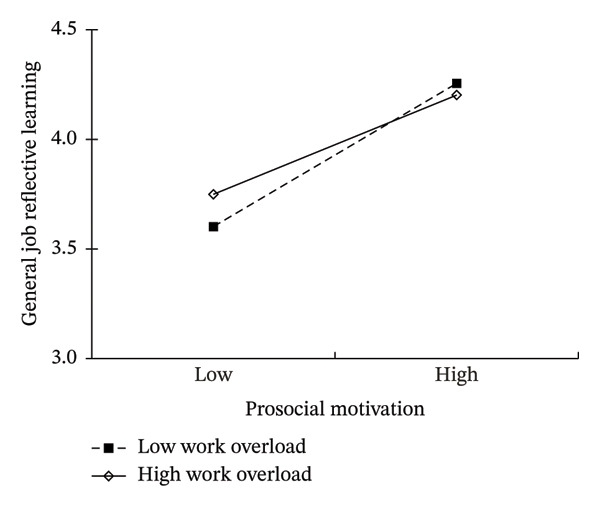
The interaction of prosocial motivation and work overload (Study 2).

The interaction between prosocial motivation and work overload on general job reflective learning was statistically significant (*B* = −0.08, *p* < 0.05), as depicted in Figure 3. Simple slope analyses further indicated that the positive relationship between prosocial motivation and general job reflective learning was attenuated under conditions of high work overload (+1 SD; simple slope = 0.29, *p* < 0.01) compared to low work overload (−1 SD; simple slope = 0.42, *p* < 0.01). These findings provide support for Hypothesis 3.

In support of Hypothesis 4, work overload significantly moderated the indirect effect of prosocial motivation on taking charge via general job reflective learning. Specifically, the index of the moderated mediation was negatively significant (index = −0.06, 95% BC CI = [−0.12, −0.00]). The indirect effect was stronger at low levels of work overload (−1 SD; indirect effect = 0.35, 95% BC CI = [0.26, 0.44]) than at high levels (+1 SD; indirect effect = 0.24, 95% CI = [0.17, 0.34]).

## 5. Study 3 Method and Results

### 5.1. Design and Participants

Study 3 employed the same procedures to recruit a cross‐national sample through Prolific. We utilized Prolific’s prescreening function to filter participants who had indicated “Nurse” under the “Employment Sector within Medicine/Healthcare” category. Prolific identified 3940 eligible nurses who had been active in the past 90 days, and we distributed our survey to this pool. Of the 501 participants who initiated the survey, 441 submitted valid responses after one participant who failed both attention‐check items, which was excluded (completion rate = 88%). Each participant was compensated GBP 3.00 for a total of 15 min of compensable time.

A total of 441 nurses completed the survey. The majority of participants were female (80.5%), with an average age of 39.33 years (SD = 11.48). Regarding educational background, 84.1% of participants held a bachelor’s degree or above. On average, participants had 13.37 years of nursing experience (SD = 10.82). Their countries of residence were primarily based in the United Kingdom (35.6%) and the United States (34.2%). Demographic characteristics of the sample are presented in Table 1.

### 5.2. Measures

#### 5.2.1. Prosocial Motivation, Work Overload, General Job Reflective Learning, and Taking Charge

We used the same scales as in Study 1 and Study 2 to measure prosocial motivation, work overload, general job reflective learning, and taking charge. Cronbach’s alphas were 0.92, 0.79, 0.88, and 0.87, respectively. We also included two open questions to understand how workload was different across nurses and how they took charge in their work context regarding voluntarily initiating constructive changes to improve work methods, policies, or procedures. Table 6 presents a summary of examples of workload differences and taking charge.

**TABLE 6:** Illustrative quotes for nurse taking charge and workload differences.

Themes	Illustrative quotes
*Nurse workload differences*	
Unit, department, and clinical setting type	“Workloads across different nursing units vary greatly. For example, critical care/emergency nurses vs. outpatient radiology nurses.”
Patient acuity and clinical complexity	“Workload varies a lot. Some nurses care for stable patients with routine tasks while others handle high acuity patients needing constant attention. Night shifts and emergencies can be much heavier due to fewer staff and sudden admissions.”
Patient volume and nurse‐to‐patient ratios	“Our workload is assigned according to geographical area, meaning that sometimes one person has many more clients than another.”
Nurse experience, seniority, and skill level	“Nurses who are more experienced and have more clinical skills tend to have more workload than those nurses who are new and less skilled.”
Job role and managerial responsibilities	“Nurses in administrative roles tend to have a heavier workload than nurses working in general care areas.”

*Nurse taking charge*	
Workflow redesign and process improvement	“I noticed a recurring delay in retrieving essential supplies for the wound care procedure. I took the initiative to reorganize the supply room and created standardized procedure kits that significantly reduced setup time and allowed for more direct patient contact.”
Documentation, digital tools, and technology adoption	“Changing the outdated process of paper PSD’s (Patient Specific Direction) to electronic, which was a cost saving (on ink/toner/paper/secretarial time for scanning) and better time management.”
Policy, protocol, and evidence‐based practice development	“I’ve helped my unit change policies that were old and outdated and helped implement new policies that have proven, evidence‐based practice. We changed our CAUTI (Catheter‐Associated Urinary Tract Infections) protocol, which directly helped the patients.”
Patient safety and quality improvement initiatives	“I championed the labeling of look‐alike drugs, sound‐alike drugs, and high alert drugs, to avoid confusion and enhance patient care.”
Handover and shift communication improvements	“I noticed inconsistencies in shift change reports that could easily lead to missed information. I proposed and helped implement a standardized SBAR (Situation, Background, Assessment, Recommendation) checklist for bedside handoffs.”

*Note: N* = 364 participants provided responses for nurse workload differences. *N* = 329 participants provided responses for nurse taking charge.

#### 5.2.2. Control Variables

Consistent with Study 1 and Study 2, we included nurses’ age, gender, job tenure, and educational level as control variables. Furthermore, we additionally controlled for proactive personality in Study 3, as prior research also found that proactive personality is positively associated with taking charge [33].

Proactive personality was measured using a 10‐item scale (*α* = 0.90) developed by Seibert et al. [36]. Sample items included “Wherever I have been, I have been a powerful force for constructive change” and “Nothing is more exciting than seeing my ideas turn into reality.” Participants responded to the items on a 7‐point Likert‐type scale (1 = *strongly disagree* to 7 = *strongly agree*).

### 5.3. Confirmatory Factor Analyses

We performed a series of CFAs to assess the distinctiveness of the four constructs in the hypothesized model. The baseline four‐factor model, including prosocial motivation, work overload, general job reflective learning, and taking charge, demonstrated a satisfactory fit to the data (*χ* ^2^ (183) = 454.02, CFI = 0.95, TLI = 0.94, SRMR = 0.05, RMSEA = 0.06). The proposed model fit the data significantly better than the alternative models: (1) a three‐factor model that combines prosocial motivation and work overload (Δ*χ* ^2^ (3) = 403.65, *p* < 0.01, CFI = 0.87, TLI = 0.85, SRMR = 0.08, RMSEA = 0.09); (2) a two‐factor model that combines prosocial motivation, work overload and general job reflective learning (Δ*χ* ^2^ (5) = 1192.97, *p* < 0.01, CFI = 0.71., TLI = 0.67, SRMR = 0.10, RMSEA = 0.13); and (3) a single‐factor model (Δ*χ* ^2^ (6) = 1915.44, *p* < 0.01, CFI = 0.56, TLI = 0.51, SRMR = 0.12, RMSEA = 0.16). These results provide strong evidence for the distinctiveness of the four variables in the hypothesized model.

### 5.4. Test of the Mediating Effects

Table 7 presents the descriptive statistics, reliabilities, and Pearson correlation coefficients among the study variables. The results in Table 8 indicate that prosocial motivation was positively related to general job reflective learning (*B* = 0.37, *p* < 0.01), thereby supporting Hypothesis 1.

**TABLE 7:** Descriptive statistics, reliabilities, and Pearson correlation coefficients (Study 3).

Variables	1	2	3	4	5	6	7	8	9
1. Age									
2. Gender	0.09								
3. Education	−0.01	0.04							
4. Job tenure	0.83^∗∗^	0.11^∗^	0.02						
5. Prosocial personality	−0.22^∗∗^	−0.07	0.08	−0.17^∗∗^	**(0.90)**				
6. Prosocial motivation	0.06	0.11^∗^	0.05	0.05	0.26^∗∗^	**(0.92)**			
7. Work overload	−0.04	0.10^∗^	0.01	−0.11^∗^	−0.11^∗^	0.05	**(0.79)**		
8. General job reflective learning	−0.04	0.02	0.08	−0.04	0.53^∗∗^	0.52^∗∗^	0.09	**(0.88)**	
9. Taking charge	−0.04	0.02	0.13^∗∗^	0.02	0.61^∗∗^	0.33^∗∗^	−0.04	0.56^∗∗^	**(0.87)**
Mean					5.18	6.31	3.74	4.11	5.19
SD					0.90	0.80	0.90	0.56	0.99

*Note: N* = 441. Cronbach’s alphas are shown in bold on the diagonal.

^∗^ *p* < 0.05.

^∗∗^ *p* < 0.01.

**TABLE 8:** Mediation model and moderated mediation model (Study 3).

	Mediation model		Moderated mediation model	
	General job reflective learning	Taking charge	General job reflective learning	Taking charge
Control variable				
Age	−0.00	−0.01	−0.00	−0.01
Gender	−0.04	0.05	−0.06	0.05
Education	0.05	0.10	0.06	0.10
Job tenure	−0.00	0.02^∗^	0.00	0.02^∗^
Prosocial personality		0.50^∗∗^		0.50^∗∗^
Independence variable				
Prosocial motivation	0.37^∗∗^	0.05	0.34^∗∗^	0.05
Mediator				
General job reflective learning		0.53^∗∗^		0.53^∗∗^
Moderator				
Work overload			0.04	
Prosocial motivation × Work overload			−0.08^∗^	
*R* ^2^	0.28	0.42	0.30	0.42

*Note: N* = 441. Bootstrap sample size = 10,000.

^∗^ *p* < 0.05.

^∗∗^ *p* < 0.01.

Furthermore, general job reflective learning was positively associated with taking charge (*B* = 0.53, *p* < 0.01). Bootstrapping results indicate that the indirect effect of prosocial motivation on taking charge through general job reflective learning was significant (indirect effect = 0.19, 95% BC CI = [0.11, 0.31]), thereby supporting Hypothesis 2.

### 5.5. Test of the Moderating Effect and the Moderated Mediation Effects

**FIGURE 4:**
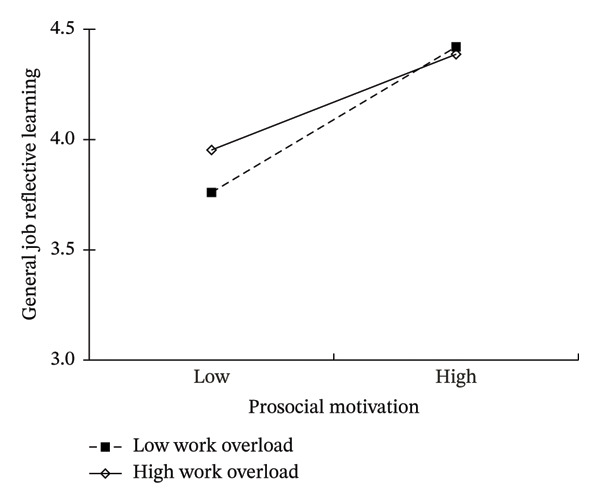
The interaction of prosocial motivation and work overload (Study 3).

The interaction between prosocial motivation and work overload in relating to general job reflective learning was statistically significant (*B* = −0.08, *p* < 0.05), as illustrated in Figure 4. Simple slope analyses further revealed that the positive relationship between prosocial motivation and general job reflective learning was weaker under conditions of high work overload (+1 SD; simple slope = 0.27, *p* < 0.01) than under low work overload (−1 SD; simple slope = 0.41, *p* < 0.01). These findings support Hypothesis 3.

In support of Hypothesis 4, work overload significantly moderated the indirect effect of prosocial motivation on taking charge via general job reflective learning. Specifically, the index of the moderated mediation was negatively significant (index = −0.04, 95% BC CI = [−0.09, −0.00]). The indirect effect was weaker at higher work overload (+1 SD; indirect effect = 0.14, 95% BC CI = [0.08, 0.24]) than at lower work overload (−1 SD; indirect effect = 0.22, 95% BC CI = [0.12, 0.33]).

## 6. Discussion

This study examined whether, why, and under what conditions prosocial motivation translates into nurses taking charge. We found that prosocial motivation was positively associated with taking charge via general job reflective learning. Moreover, under higher work overload, the positive association between prosocial motivation and reflective learning was weaker, and the indirect effect on taking charge was less pronounced. Below, we outline the implications, limitations, and directions for future research.

### 6.1. Theoretical Implications

First, this research extends the prosocial motivation literature by showing that nurses’ desire to benefit others can motivate proactive efforts to challenge the status quo and drive systemic improvements in the healthcare environment. In increasingly complex and uncertain environments, organizations need employees not only to perform assigned tasks effectively but also to take proactive steps in how work is executed [23]. Unlike helping—an affiliative, other‐oriented behavior aimed at maintaining the status quo by promoting and supporting existing work processes and relationships [37]—taking charge is a challenging, proactive, and other‐oriented behavior aimed at changing the status quo by questioning and improving existing processes and relationships. As such, taking charge holds unique value in this context but typically involves higher risk [12, 13, 38]. These risks are amplified in healthcare settings, where change‐oriented initiatives are often reserved for formal leaders (e.g., charge nurses) [14, 15], making frontline change efforts more precarious. Using a self‐regulation framework to investigate whether, why, and under what conditions nurses convert prosocial motivation into taking charge, we identify general job reflective learning as a mechanism and work overload as a boundary condition, thereby addressing calls to test mechanisms for the effects of prosocial motivation beyond helping [39]. By shifting attention from formal leaders to frontline nurses as initiators of change [15], this study also highlights the microfoundations of organizational improvement in clinical settings.

Second, this research enriches the integration of prosocial motivation and proactivity literature by specifying general job reflective learning as a critical, yet understudied, cognitive mechanism that bridges prosocial motivation and proactive behavior. Although taking charge is inherently risky [12, 13], prior research often assumes that a desire to benefit others will spur employees to take charge despite these risks [38, 40], overlooking that taking charge involves calculated and deliberate decision‐making [8]. Applying self‐regulation theory [17], we introduce and empirically validate general job reflective learning as the essential self‐regulatory step that translates prosocial motivation into taking charge. By revealing this informed, risk‐aware learning process, the study answers calls to investigate how employees act proactively in “wise” ways [23], which is especially important in high‐stakes clinical contexts.

Third, this research contributes to the workload literature by highlighting work overload not merely as a resource‐draining stressor but as a cognitive inhibitor that constrains personal and organizational development. While existing research has largely treated work overload as a stressor that is associated with diminished personal resources and poorer well‐being [25, 41], its profound role in personal growth and organizational development remains underexplored. Using a self‐regulatory perspective, we show that overload not only drains resources but also interferes with the reflective process required for learning and improvement. Specifically, we found that under high work overload, prosocial motivation was less likely to translate into general job reflective learning, which in turn constrains taking charge. This reveals a critical cognitive pathway through which workload dampens the translation of prosocial motivation into personal development and proactive organizational improvement efforts. By examining the interplay between prosocial motivation and work overload, this research provides a microfoundation for when and why mission‐driven organizations struggle to sustain continuous improvement—an issue of particular importance in clinical settings.

### 6.2. Practical Implications

This research offers actionable guidance for healthcare administrators and policymakers seeking to foster a culture of proactive improvement and empower frontline nurses to take charge.

First, because prosocial motivation has a positive indirect effect on taking charge, organizations should actively cultivate it by reinforcing the ethical and mission‐driven aspects of nursing. Strategies include systematically sharing positive patient feedback, highlighting the direct impact of nursing work on patient welfare, and designing work environments that make positive outcomes salient. Strengthening nurses’ desire to benefit others can initiate self‐regulatory efforts that drive constructive change.

Second, because general job reflective learning mediates the link between prosocial motivation and taking charge, organizations should recognize and support reflective learning as a critical professional activity. Administrators can allocate protected time for structured reflection—such as postaction reviews, systematic debriefings, and peer learning sessions. Treating reflective learning as an essential investment rather than a discretionary luxury enhances nurses’ strategic capability and confidence, thereby enabling them to take charge successfully.

Third, given that the positive relationship between prosocial motivation and general job reflective learning is weaker under high work overload, administrators must prioritize resource management. Ensuring adequate staffing, optimizing patient‐to‐nurse ratios, and streamlining administrative burdens can reduce overload, protect energy and psychological space, and allow prosocially motivated nurses to engage in reflective planning and sustain taking charge.

### 6.3. Limitations and Recommendations for Future Research

First, Study 1 was conducted in a single hospital in China, which raises potential concerns regarding the generalizability of our findings. To address this issue, we conducted Study 2 and Study 3 using the Prolific platform to collect data from a broader, multinational nursing workforce. Nevertheless, we encourage future studies to continue to test our theoretical model across other specific cultural or organizational contexts to fully map its applicability.

Second, we collected data in Study 2 and Study 3 via Prolific, an online crowdsourcing platform widely adopted in behavioral and organizational research. Although crowdsourcing platforms typically offer the benefit of providing a large and diverse participant pool, they also present challenges such as high dropout rates [42]. In both Studies 2 and 3, the dropout rates are low. Nevertheless, future research should seek to employ alternative data collection approaches to further validate our findings.

Third, we view reflective learning as a self‐regulatory process and focus on its general form. Future research can unpack distinct forms of reflection and their implications for proactivity. For instance, employees may reflect on both successes (positive task experiences and favorable work relationships) and failures (negative task experiences and unfavorable work relationships) [29]. Reflection on failures may serve as a stronger driver of high‐risk behaviors like taking charge because it often involves deeper cognitive processing to identify systemic flaws and internal causes, whereas reflection on successes may enhance self‐efficacy and reinforce existing effective practices. Future studies can test whether reflective learning from successes and failures differentially mediate the relationship between prosocial motivation and taking charge, yielding a more granular account of how prosocial employees convert motive into action.

Fourth, while we identify work overload as a contextual factor that attenuates the motivational process, future research should explore resource‐enhancing boundary conditions. Supervisor support, for example, is an organizational resource that can buffer the negative effects of depletion [43]. Future work could examine whether strong supervisor support strengthens the link between prosocial motivation and general job reflective learning, particularly under high workload.

## 7. Conclusion

Using a self‐regulatory perspective, this study investigates whether, why, and under what conditions nurses convert prosocial motivation into taking charge. The findings show that prosocial motivation can move nurses beyond routine helping to pursue constructive change and identify general job reflective learning as the critical self‐regulatory process that channels prosocial motivation into taking charge. The study also underscores how work overload hinders this process, demonstrating that prosocial motivation alone may be insufficient when nurses lack the resources required for self‐regulation. These insights provide theoretical contributions and practical guidance for healthcare administrators seeking to cultivate a culture in which nurses are enabled and equipped to lead necessary improvements by fostering prosocial motivation, supporting reflective learning, and reducing excessive workload.

## Funding

This work was supported by the Zhejiang Provincial Health Commission project in China (2023KY118).

## Disclosure

All authors read and approved the final manuscript.

## Conflicts of Interest

The authors declare no conflicts of interest.

## Supporting Information

Additional supporting information can be found online in the Supporting Information section.

## Supporting information


**Supporting Information** STROBE‐checklist.

## Data Availability

The data that support the findings of this study are available on request from the corresponding author. The data are not publicly available due to privacy or ethical restrictions.

## References

[1] World Health Organization (WHO) , State of the World’s Nursing Report 2025, 2025, https://iris.who.int/server/api/core/bitstreams/a4173924-a18f-49b6-8bd1-9c2a4a098980/content.

[2] Grant A. M. and Sumanth J. J. , Mission Possible? the Performance of Prosocially Motivated Employees Depends on Manager Trustworthiness, Journal of Applied Psychology. (2009) 94, no. 4, 927–944, 10.1037/a0014391. 19594235

[3] Grant A. M. , Does Intrinsic Motivation Fuel the Prosocial Fire? Motivational Synergy in Predicting Persistence, Performance, and Productivity, Journal of Applied Psychology. (2008) 93, no. 1, 48–58, 10.1037/0021-9010.93.1.48. 18211134

[4] Riggio R. E. and Taylor S. J. , Personality and Communication Skills as Predictors of Hospice Nurse Performance, Journal of Business and Psychology. (2000) 15, no. 2, 351–359, 10.1023/A:1007832320795.

[5] Shen L. , Fei X. , Zhou Y. , Wang J. , Zhu Y. , and Zhuang Y. , The Effect of Felt Trust from Patients Among Nurses on Attitudes Towards Nursing Service Delivery, Journal of Advanced Nursing. (2022) 78, no. 2, 404–413, 10.1111/jan.14973. 34363632

[6] Mayo A. T. , Myers C. G. , and Sutcliffe K. M. , Organizational Science and Health Care, The Academy of Management Annals. (2021) 15, no. 2, 537–576, 10.5465/annals.2019.0115.

[7] Thirumalai S. , Devaraj S. , and Browning T. R. , Uncertainty in Healthcare Operations: How Hospitals Weather the Perfect Storm, Journal of Operations Management. (2024) 70, no. 8, 1194–1212, 10.1002/joom.1327.

[8] Morrison E. W. and Phelps C. C. , Taking Charge at Work: Extrarole Efforts to Initiate Workplace Change, Academy of Management Journal. (1999) 42, no. 4, 403–419, 10.5465/257011.

[9] American Nurses Association , Nursing: Scope and Standards of Practice, 2021, 4th edition, American Nurses Association.

[10] National Academies of Sciences and Engineering and Medicine , The Future of Nursing 2020-2030: Charting a Path to Achieve Health Equity, 2021, The National Academies Press. 34524769

[11] Institute of Medicine , The Future of Nursing: Leading Change, Advancing Health, 2011, National Academies Press, Washington, DC. 24983041

[12] Fuller J. B. , Marler L. E. , Hester K. , and Otondo R. F. , Leader Reactions to Follower Proactive Behavior: Giving Credit when Credit is due, Human Relations. (2015) 68, no. 6, 879–898, 10.1177/0018726714548235.

[13] Zhang M. J. , Law K. S. , and Wang L. , The Risks and Benefits of Initiating Change at Work: Social Consequences for Proactive Employees Who Take Charge, Personnel Psychology. (2021) 74, no. 4, 721–750, 10.1111/peps.12423.

[14] Duygulu S. and Kublay G. , Transformational Leadership Training Programme for Charge Nurses, Journal of Advanced Nursing. (2011) 67, no. 3, 633–642, 10.1111/j.1365-2648.2010.05507.x. 21077934

[15] Sherman R. O. and Eggenberger T. , Taking Charge: what Every Charge Nurse Needs to Know, Nurses First. (2009) 2, no. 4, 6–10, https://emergingrnleader.com/wp-content/uploads/2012/06/NursesFirst09August.pdf#page=6.

[16] Grant A. M. and Mayer D. M. , Good Soldiers and Good Actors: Prosocial and Impression Management Motives as Interactive Predictors of Affiliative Citizenship Behaviors, Journal of Applied Psychology. (2009) 94, no. 4, 900–912, 10.1037/a0013770. 19594233

[17] Zimmerman B. J. , Boekaerts M. , Pintrich P. R. , and Zeidner M. , Attaining Self-Regulation: a Social-Cognitive Perspective, Handbook of Self-Regulation, 2000, Academic Press, San Diego, 13–39.

[18] Krogstie B. R. , Prilla M. , and Pammer V. , Hernández-Leo D. et al., Understanding and Supporting Reflective Learning Processes in the Workplace: the CSRL Model, Scaling up Learning for Sustained Impact, Lecture Notes in Computer Science, 2013, 8095, Springer, 151–164.

[19] Schunk D. H. and Zimmerman B. J. , Reynolds W. M. , Miller G. E. , and Weiner I. B. , Self-Regulation and Learning, Handbook of Psychology: Educational Psychology, 2013, 2nd edition, John Wiley & Sons, 45–68.

[20] Peltier J. W. , Hay A. , and Drago W. , The Reflective Learning Continuum: Reflecting on Reflection, Journal of Marketing Education. (2005) 27, no. 3, 250–263, 10.1177/0273475305279657.

[21] Bai Y. , Wang J. , Chen T. , and Li F. , Learning from Supervisor Negative Gossip: the Reflective Learning Process and Performance Outcome of Employee Receivers, Human Relations. (2020) 73, no. 12, 1689–1717, 10.1177/0018726719866250.

[22] Parker S. K. , Bindl U. K. , and Strauss K. , Making Things Happen: a Model of Proactive Motivation, Journal of Management. (2010) 36, no. 4, 827–856, 10.1177/0149206310363732.

[23] Parker S. K. , Wang Y. , and Liao J. , When is Proactivity Wise? A Review of Factors that Influence the Individual Outcomes of Proactive Behavior, Annual Review of Organizational Psychology and Organizational Behavior. (2019) 6, no. 1, 221–248, 10.1146/annurev-orgpsych-012218-015302.

[24] Muraven M. and Baumeister R. F. , Self-Regulation and Depletion of Limited Resources: Does Self-Control Resemble a Muscle?, Psychological Bulletin. (2000) 126, no. 2, 247–259, 10.1037/0033-2909.126.2.247. 10748642

[25] Lee S. E. , Seo J. K. , and Macphee M. , Effects of Workplace Incivility and Workload on Nurses’ Work Attitude: the Mediating Effect of Burnout, International Nursing Review. (2024) 71, no. 4, 1080–1087, 10.1111/inr.12974. 38661534 PMC11600474

[26] Kohnen D. , De Witte H. , Schaufeli W. B. et al., Key Drivers of Nurse Burnout and Work Engagement in Europe: a Cross-Sectional Dominance Analysis, International Journal of Nursing Studies. (2026) 173, 10.1016/j.ijnurstu.2025.105251. 41175851

[27] Yoon J. , Han N. C. , and Seo Y. J. , Sense of Control Among Hospital Employees: an Assessment of Choice Process, Empowerment, and Buffering Hypotheses, Journal of Applied Social Psychology. (1996) 26, no. 8, 686–716, 10.1111/j.1559-1816.1996.tb02739.x.

[28] Grant A. M. and Berry J. W. , The Necessity of Others is the Mother of Invention: Intrinsic and Prosocial Motivations, Perspective Taking, and Creativity, Academy of Management Journal. (2011) 54, no. 1, 73–96, 10.5465/amj.2011.59215085.

[29] Li F. , Chen T. , Chen N. Y. F. , Bai Y. , and Crant J. M. , Proactive yet Reflective? Materializing Proactive Personality into Creativity Through Job Reflective Learning and Activated Positive Affective States, Personnel Psychology. (2020) 73, no. 3, 459–489, 10.1111/peps.12370.

[30] Jin M. , Qian R. , Wang J. et al., The Mediating Effect of Coping Styles Between Emergency Capacity and Mental Workload Among Clinical Nurses: a Cross‐Sectional Study, International Nursing Review. (2024) 71, no. 4, 1121–1129, 10.1111/inr.12985. 38899768

[31] Moghadam K. N. , Chehrzad M. M. , Masouleh S. R. et al., Nursing Workload in Intensive Care Units and the Influence of Patient and Nurse Characteristics, Nursing in Critical Care. (2021) 26, no. 6, 425–431, 10.1111/nicc.12548. 32954619

[32] Yang H. , van der Heijden B. , Shipton H. , and Wu C. , The Cross-Level Moderating Effect of Team Task Support on the Nonlinear Relationship Between Proactive Personality and Employee Reflective Learning, Journal of Organizational Behavior. (2022) 43, no. 3, 483–496, 10.1002/job.2572.

[33] Fuller J. B. and Marler L. E. , Change Driven by Nature: a Meta-Analytic Review of the Proactive Personality Literature, Journal of Vocational Behavior. (2009) 75, no. 3, 329–345, 10.1016/j.jvb.2009.05.008.

[34] Aiken L. S. and West S. G. , Multiple Regression: Testing and Interpreting Interactions, 1991, Sage Publications.

[35] Preacher K. J. , Rucker D. D. , and Hayes A. F. , Addressing Moderated Mediation Hypotheses: Theory, Methods, and Prescriptions, Multivariate Behavioral Research. (2007) 42, no. 1, 185–227, 10.1080/00273170701341316. 26821081

[36] Seibert S. E. , Crant J. M. , and Kraimer M. L. , Proactive Personality and Career Success, Journal of Applied Psychology. (1999) 84, no. 3, 416–427, 10.1037/0021-9010.84.3.416. 10380421

[37] Liao H. , Su R. , Ptashnik T. , and Nielsen J. , Feeling Good, Doing Good, and Getting Ahead: a Meta-Analytic Investigation of the Outcomes of Prosocial Motivation at Work, Psychological Bulletin. (2022) 148, no. 3–4, 158–198, 10.1037/bul0000362.

[38] Xu A. J. , Loi R. , and Chow C. W. C. , Why and when Proactive Employees Take Charge at Work: the Role of Servant Leadership and Prosocial Motivation, European Journal of Work & Organizational Psychology. (2022) 31, no. 1, 117–127, 10.1080/1359432X.2021.1934449.

[39] Bolino M. C. and Grant A. M. , The Bright Side of Being Prosocial at Work, and the Dark Side, Too: a Review and Agenda for Research on Other-Oriented Motives, Behavior, and Impact in Organizations, The Academy of Management Annals. (2016) 10, no. 1, 599–670, 10.5465/19416520.2016.1153260.

[40] Cai Z. , Huo Y. , Lan J. , Chen Z. , and Lam W. , When Do Frontline Hospitality Employees Take Charge? Prosocial Motivation, Taking Charge, and Job Performance: the Moderating Role of Job Autonomy, Cornell Hospitality Quarterly. (2018) 60, no. 3, 237–248, 10.1177/1938965518797081.

[41] Bakker A. B. and Demerouti E. , Job Demands–Resources Theory: Taking Stock and Looking Forward, Journal of Occupational Health Psychology. (2017) 22, no. 3, 273–285, 10.1037/ocp0000056. 27732008

[42] Aguinis H. , Villamor I. , and Ramani R. S. , MTurk Research: Review and Recommendations, Journal of Management. (2021) 47, no. 4, 823–837, 10.1177/0149206320969787.

[43] Luo W. , Wang J. L. , Chen T. et al., How the Approaches of Managing Conflict with Patients Affect Nurse Emotional Exhaustion and Life Satisfaction: a Time-Lagged Three-Wave Survey, Journal of Advanced Nursing. (2025) 81, no. 6, 3025–3035, 10.1111/jan.16455. 39304314

